# Plant-Based Polyphenols: Anti-*Helicobacter pylori* Effect and Improvement of Gut Microbiota

**DOI:** 10.3390/antiox11010109

**Published:** 2022-01-04

**Authors:** María Guerra-Valle, Patricio Orellana-Palma, Guillermo Petzold

**Affiliations:** 1 Programa de Doctorado en Ingeniería de Alimentos, Universidad del Bío-Bío, Av. Andrés Bello 720, Chillan 3780000, Chile; maria.guerra1601@egresados.ubiobio.cl; 2 Laboratorio de Crioconcentración, Departamento de Ingeniería en Alimentos, Universidad del Bío-Bío, Av. Andrés Bello 720, Chillan 3780000, Chile; 3 Departamento de Ingeniería en Alimentos, Facultad de Ingeniería, Universidad de La Serena, Av. Raúl Bitrán 1305, La Serena 1700000, Chile; 4 Grupo de Crioconcentración de Alimentos y Procesos Relacionados, Universidad del Bío-Bío, Av. Andrés Bello 720, Chillan 3780000, Chile

**Keywords:** *Helicobacter pylori*, gut microbiota, bioactive compounds

## Abstract

*Helicobacter pylori* (*H. pylori*) infection affects more than half of the world’s population, and thus, about 10 to 20% of people with *H. pylori* suffer from peptic ulcers, which may ultimately lead to gastric cancer. The increase in antibiotic resistance and susceptibility has encouraged the search for new alternative therapies to eradicate this pathogen. Several plant species are essential sources of polyphenols, and these bioactive compounds have demonstrated health-promoting properties, such as the gut microbiota stimulation, inflammation reduction, and bactericidal effect. Therefore, this review aims to discuss the potential effect of plant-based polyphenols against *H. pylori* and their role in the gut microbiota improvement.

## 1. Introduction

*Helicobacter pylori* (*H. pylori*) is a Gram-negative spiral bacterium that has long been recognized by its ability to colonize chronically the stomach lining and the upper section of the intestine, generating gastritis, gastroduodenal ulcers and gastric carcinoma [1]. Specifically, *H. pylori* establishes the optimal environmental conditions to survive short acidic exposures, since it produces two enzymes, urease and carbonic anhydrase (CA), where the urease converts the host urea into ammonia and carbon dioxide (CO_2_), while CA catalyzes the transformation of CO_2_ into bicarbonate (HCO_3_-). Hence, both reactions allow to increase the pH levels. Thereby, the host’s immune response is affected by the constant inflammation of the epithelial cells, causing the adaption and survival of the *H. pylori* over time in the local environmental conditions [2,3]. Furthermore, *H. pylori* continuously moves along the host tissues through chemotaxis (directed motility) by controlling flagellar rotation, where the bacterial cells travel toward advantageous environments or away from disadvantageous environments [4,5,6,7], being this movement key to colonize from the lumen to the mucus layer, overlying the epithelium due to less acidic conditions [8]. Moreover, another factor that may influence *H. pylori* colonization is the configuration of the stomach, since the human stomach is a heterogeneous and complex system, composing of various kinds of regions that differ in cellular composition and conditions, where the human stomach has three glandular regions: fundus, corpus, and antrum [9].

**Figure 1:**
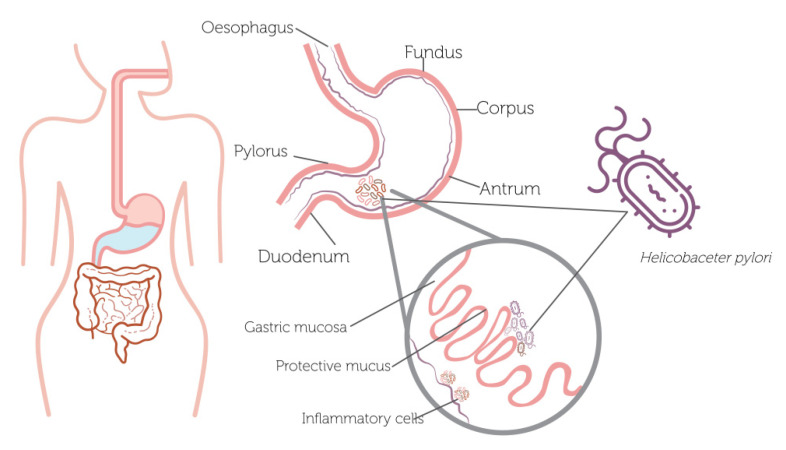
*Helicobacter pylori* colonization of the human stomach.

*H. pylori* infection affects more than half of the world population, and it plays a crucial role in gastric diseases [10]. This pathogen causes gastroduodenal inflammation that can lead to atrophic gastritis, gastric ulcers, and, finally, gastric cancer (one of the major causes of mortality worldwide, according to the National Comprehensive Cancer Network) [11]. As shown in Figure 1, *H. pylori* generally proliferates in the upper section of the intestine and gastric mucosa, triggering an inflammatory process.

According to Sjomin et al. [12], the global prevalence of *H. pylori* is similar between genders, with 42.7% in females and 46.3% in males [13], increasing with risks factors on eating habits and lifestyle behaviors such as poor hand hygiene, low frequency of hand-washing practice, low consumption of fruits and vegetables, and high consumption of fried foods [12]. Hence, around 20% of the world population infected with *H. pylori* suffers peptic ulcers, which ultimately produce gastric cancer, and thus, studies have indicated that 75% of gastric cancer can be attributed to *H. pylori* infection [4,14].

In the same way, the most common treatment against *H. pylori* infection is the combination of two antibiotics and a proton pump inhibitor administered twice a day, and it presents high effectivity (≈90%). However, the treatment produces metabolic side effects such as mitochondrial damage and hypoprothrombinemia [15,16]. In addition, the human genetic susceptibility to antibiotics, the various mechanisms of *H. pylori* to resist gastric acidity conditions, and the globally observed increase in antibiotic resistance of *H. pylori* have led researchers to seek and propose effective and novel alternative therapies against this pathogen through the use of different bioactive components.

Thus, in recent times, polyphenols and phytochemicals from plants, fruits, and vegetables, have demonstrated interesting antimicrobial properties, suggesting the possibility that bioactive compounds can assist in the prevention and treatment of microbial pathogens. Thereby, several studies have assessed the role of bioactive compounds as antibacterial properties, and ways to modulate the gut microecosystem to promote the population of probiotics, and thus, favoring effective prevention and treatments against bacterial infections [17,18,19].

Therefore, this review aims to discuss the therapeutic effects of plant-based polyphenols against *H. pylori* and the potential role to improve the gut microbiota in human metabolic health.

## 2. Healthy Effects of Polyphenols and Antioxidants

Polyphenols are natural bioactive compounds derived from plants, fruits, and vegetables. These bioactive compounds have different phenolic hydroxyl groups in their structure, and thus, it is possible to identify phenolic acids, tannins, carotenoids, flavonoids, anthocyanins, and proanthocyanidins, among others, where the molecules present a wide variety of concentrations in the foods [20]. Thereby, polyphenols display a myriad of health-beneficial properties on the human body, since these components have demonstrated different medicinal and nutraceutical properties, helping in the gastrointestinal digestion [21], reducing the blood lipid levels [22], improving the body immunity [23], and in addition, polyphenols present neuroprotective, cardioprotective, anti-inflammatory, anti-diabetic, anti-carcinogenic, and anti-ageing effects, among other health improvements [24,25,26]. Nonetheless, despite all advantages of polyphenols, these components are very bitter and astringent tastes, making it difficult to incorporate into food or model foods [27], and also, polyphenols are unstable under certain conditions such as exposure to light, presence of oxygen and enzymes, high temperatures, and pH changes, weakening their activity [28,29,30].

An important point, studies on the potential prebiotic effect of polyphenols on the composition and function of the gut microbiota, gut permeability, gut-derived proinflammatory stimuli, have increased considerably, and the results have been linked to the high production of anti-inflammatory molecules such as interleukin-4 (IL-4), interleukin-10 (IL-10), interleukin-13 (IL-13), and adiponectin [31,32,33,34]. Moreover, dietary polyphenols can contribute substantially to reduce the frequency of oxidative stress-induced damage triggered by inflammation or infectious process, and thus, it plays a vital role in chemoprevention through different action mechanisms such as nuclear factor erythroid 2-related factor 2 (Nrf2) and Nrf2-antioxidant response element (ARE) pathway, and phase I and phase II detoxifying enzymes [35]. Specifically, Nrf2-Are is an essential intermediary, since it induces cellular response against oxidative stress, a condition due to either a mechanism of aggressive cytotoxicity level, producing more reactive oxygen species (ROS) or the cellular incapacity to remove the reaction of reactive oxidant [36]. Besides, for phase I detoxifying enzymes, it interacts with nucleophilic groups, causing mutagenic gashes. Then, the products of phase I are purified through phase II detoxifying enzymes, inactivating ROS and detoxifying carcinogens. Thus, phase II enzymes catalyze electrophiles conjugation along with metabolites (xenobiotics and endogenous) of oxidative stress-induced lipid peroxidation [35]. Thereby, there is an association between diets with high polyphenol and antioxidant contents, allowing minimal toxicity of healthy tissues due to the reduction of oxidative stress and the protection of extracellular and intracellular DNA [37,38].

During the process of colonization of the intestinal epithelium, *H. pylori* triggers oxidant-sensitive transcription factors that cause tissue damage at the molecular and cellular levels [39], and consequently the activation of nuclear factor kappa-light chain-cancer-activated B cells (NF-kB), which induces the expression of the interleukin-8 (IL-8) gene [40,41,42]. In addition, *H. pylori* also activate nicotinamide adenine dinucleotide dinucleotide phosphate (NADPH) oxidase, which is a major source of ROS, also the cyclooxygenase (COX), nitric oxide (NO) and reactive nitrogen species (RNS) [43]. Mitochondria are susceptible to attack by ROS, and thus, these dysfunctional mitochondria drive the production of inflammatory cytokines [44].

The main attribute of polyphenols and their metabolites is their antioxidant action by targeting immune cells activating different signaling pathways that alter interleukins, cyclooxygenase, nitric oxide synthase and other inflammatory responses [45] caused by diseases and infections, such as *H. pylori*. Some polyphenols affect the NF-kB pathway by inhibiting the phosphorylation of kinases; preventing the translocation and transcription of proinflammatory mediators, also hindering the interaction between NF-kB and its predicted DNA [46]. Each type of polyphenol has a particular antioxidant mechanism; it is believed that the antioxidant activity of phenolic acids lies in their ability to scavenge radicals by donating hydrogen atoms [47]. In the case of stilbenoids, their antioxidant action is attributed to the presence of a hydroxyl in the ortho-position, which allows them to scavenge superoxide radicals [48]. Likewise, flavonoids have the ability to counteract the NF-kB signaling pathway and prevent the inflammatory effects of NO [46]; the inhibitory effects of flavonoids on the colonic expression of one of the COX isoforms (inducible COX-2) have also been described [49]. Nrf2 and NF-kB signaling pathways can be modulated by anthocyanins, catechins and ellagitannins; also anthocyanins can reduce COX-2 and NO and ellagitannins decrease IL-8 secretion [46]. Therefore, the action of polyphones in the management of *H. pylori* infection is by modulating inflammatory signals and factors that induce oxidative stress and thus the appearance of free radicals associated with further tissue damage.

Specifically, the studies on bioactive compounds, derived from foods, have grown exponentially since the mid-1990s, due to their beneficial effects as antimicrobial agents, and in addition, bioactive compounds have received growing attention due to the antibiotic resistance of pathogenic bacteria [50]. For example, a meta-analysis study was conducted to indicate the beneficial effects on human health of polyphenol-rich foods such as apples, wine, tea, and berries. This study emphasized that the doses of polyphenol intake affects the abundance of human gut microbiota, where the intake of polyphenols changed the gut microbiota of human subjects, since it stimulated the presence of *Lactobacillus* and *Bifidobacterium* species, and in turn, the polyphenols limited the presence *Clostridium* species. Thus, the polyphenols intake between 396–540 mg/day maintains a positive balance in the gut microbiota composition due to the increase in health-promoting species [51]. Moreover, recent works have emphasized the influence of dietary patterns on polyphenols daily intake and its effect on the gut microbiota composition, since a study in a U.S. cohort concluded that the diet presents between 498–662 mg/day [52], while Mediterranean dietary pattern has an average of 1171 mg/day [53], and Atlantic Diet provides 1011–1284 mg/day of polyphenols [54]. Therefore, each diet has a different effect in the body’s immune system against infectious agents such as viruses and bacteria (*H. pylori*) [55].

In this context, authors have indicated that *H. pylori* infection affects oxidative stress status of the host [56,57,58]. However, the consumption of dietary antioxidants can be effective to reduce negative aspects of *H. pylori* [59]. In this way, Judaki et al. [60] reported a significant reduction in the amount of oxidative DNA damage and apoptosis in *H. pylori* positive individuals after consuming curcumin (700 mg three times daily) during three months. Also, Jones et al. [61] demonstrated that capsaicin inhibits the growth of *H. pylori*, since capsaicin has been recognized as a potential anti-inflammatory agent due to the production of IL-8 in the gastric epithelium of *H. pylori*-positive individuals [62]. Similarly, Yanagawa et al. [63] showed that epigallocatechin-3-gallate (from green tea) increased the antibacterial activity of an antibiotic therapy, with high efficiency against *Helicobacter pylori* growth in vitro.

However, despite these advantages of polyphenols for human health, there are some concerns about polyphenol consumption, since the fortification and supplementation from polyphenol extracts can substitute fruit and vegetable intake, but these extracts may not have the same health benefits as the fresh fruits and vegetables due to different interactions between compounds after the extraction methods [64]. Silva and Pogačnik [65] have recognized the need to study any relationship between the overconsumption of polyphenol intake and the effect on the gut microbiota due to the accumulation of different molecules in the organism.

## 3. Polyphenols and Gut Microbiota

### 3.1. Biotransformation of Polyphenols by Gut Microbiota

Polyphenols present a low absorption rate in the human gut, where the colon has highest percentage of polyphenols absorption [66]. From this, multiple investigations have evaluated the role of phenolic compounds to regulate the intestinal microecosystem and to promote the growth of healthy bacteria such as *Akkermansia*, *Faecalibacterium*, *Lactobacillus*, *Bifidobacterium* and *Enterococcus* spp., since these microorganisms have been recognized for their anti-inflammatory and immuno-regulating effects, and thus, these species can be useful against infectious pathogens [67,68,69].

As mentioned above, polyphenols can be hydrolyzed by enzymes or metabolized by the gut microbiota. Thereby, these compounds differ from those in fresh or processed foods, since the polyphenols hydrolyzed (or metabolized) can reach the bloodstream, tissues, and brain, of the host [70]. In the same way, the chemical structure of polyphenols can be related to their bioavailability. Thus, polyphenols commonly consumed in the diet are esters, glycosides, or polymers, but these compounds cannot be absorbed in their original form [64]. Therefore, gut microbiota plays a vital role in the biotransformation of bioactive compounds, especially polyphenols, where 5–10% can be absorbed in the small intestine, and the remaining percentage can accumulate in the large intestine [71,72]. Hence, the microbiota produces decomposition of polyphenol structures into low-molecular-weight phenolic compounds, and they all have health positive effects on consumers, but the biotransformation varies significantly among individuals and populations [73]. Concretely, the bioaccessibility of phenolic compounds can vary significantly. For example, the anthocyanins have a bioaccessibility close to 5–10%, i.e., approximately 90–95% of polyphenols reach the colon [74]. Thus, the biotransformation by gut microbiota mainly involves the interaction of *Bacteroides*, *Bifidobacterium*, *Clostridium*, *Fusobacterium*, *Lactobacillus* and *Peptostreptococus* species [75]. Table 1 displays the biotransformation of polyphenols by gut microbiota and main metabolites.

### 3.2. Metabolism of Dietary Polyphenols: Role of the Microbiota

**Figure 2:**
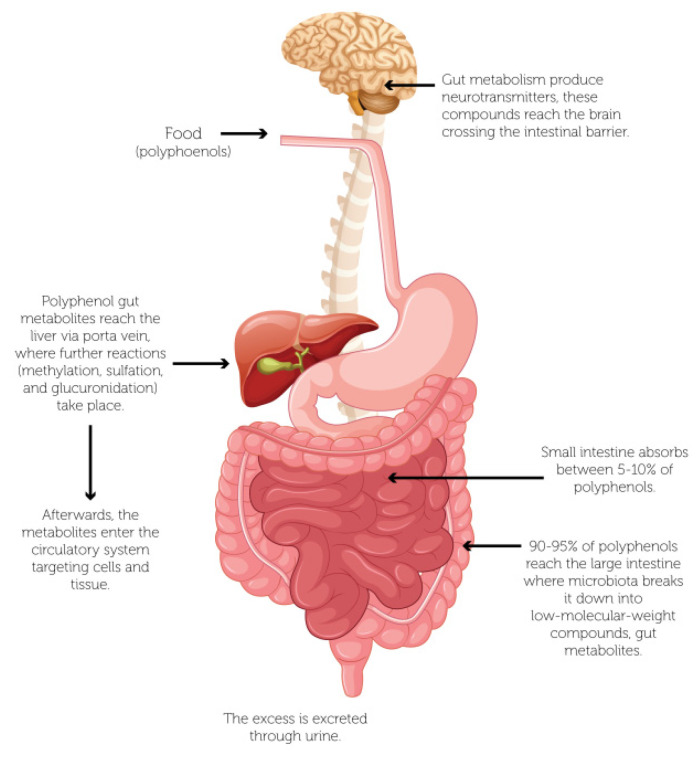
*Helicobacter pylori* colonization of the human stomach.

Polyphenols can influence the function and composition of the microbiota, since the microbiota generates metabolites (mainly aromatic and phenolic acids) through interaction with polyphenols, and the biotransformation process occurs mainly in the large intestine [63]. Figure 2 shows the biotransformation of polyphenols from food in the gastrointestinal tract, where the microbiota breaks the polyphenols into low-molecular-weight compounds through hydroxylation, isomerization, dehydrogenation, glycosylation decarboxylation, and methylation, among other chemical reactions. Then, the metabolites are absorbed in the small intestine and reach the liver through the portal vein, where other reactions are carried out by several methods such as methylation, sulfation, and glucuronidation. Hence, these metabolites enter the circulatory system and transferred to remote cells and tissues [72,77,78].

In vitro assays indicate that polyphenols may exert prebiotic-like effects on the intestinal microflora, promoting the growth [79]. Specifically, an in vitro study on tea polyphenols, it revealed the potential role of polyphenols to improve the profusion of *Lactobacillus*, *Enterococcus* and *Bifidobacterium* spp. [80,81]. Tomás-Barberán et al. [18] concluded that polyphenols could modulate the growth of *Roseburia*, *Akkermansia* and *Faecalibacterium* spp. Thereby, there is synergy between dietary polyphenols and gut microbiota, and thus, the gut microbiota can transform polyphenols into secondary metabolites, making them more bioavailable, and with highly beneficial effects to the consumers. Therefore, while polyphenols can modify the microbiota, the growth of pathogens can be prevented [82].

## 4. Anti-*Helicobacter pylori* Effects of Plant-Based Polyphenols

Various studies have highlighted the potential role of polyphenols against the adverse consequences of *H. pylori* infection [83]. However, the polyphenols do not totally eradicate *H. pylori* from the host, but these components can significantly reduce the negative effects (bacterial colonization, inflammatory responses and mucosal atrophy) of the infection, and in turn, the polyphenols allow to reduce the dose of antibiotics given to the patient, since the interaction polyphenols/antibiotics have demonstrated synergistic effects on *H. pylori* action [84]. Thus, ellagic acid has shown promising effects, since Chung [85] demonstrated excellent results with increased dose of ellagic acid on the arylamine N-acetyltransferase (NAT) activity in *H. pylori* cytosols, with different percentages of 2-aminofluorene and p-aminobenzoic acid acetylation in relation to the concentration of ellagic acid. Similarly, Martini [86] showed that isolated polyphenols such as ellagic acid, kaempferol, gallic acid and quercetin 3-O-β-D-glucopyranoside had different bactericidal effects against two *H. pylori* strains (CagA^+^ strain 10 K and CagA^−^ strain G21), suggesting that the factors of time of exposure and concentration are responsible for a correct saturation of the CagA strains, and in addition, the individual polyphenols inactivated the *H. pylori* ion pumps (the flux of copper and metal cations through membranes regulated by enzymes). Zhang et al. [87] showed the inhibition of *H. pylori* infection through resveratrol, since this component decreased the *H. pylor*i-induced mRNA transcription and protein expression levels of inducible nitric oxide synthase (iNOS) and IL-8, and it decreased the urease activity by ≈90%. Moreover, resveratrol increased the activity of heme oxygenase-1 (HO-1), an antioxidant enzyme with therapeutic target against oxidative stress and gastrointestinal diseases, and Nrf2, a transcriptional regulator of HO-1, and in turn, a cellular sensor of oxidative stress and it induces expression of cytoprotective genes, reducing the damage of ROS. Lee et al. [88] indicated that epigallocatechin inhibited the glycosylation of *H. pylori*-induced toll-like receptor 4 (TLR-4), and also, the component inhibited the urease enzyme, diminishing the damage on DNA and gastric mucosa cytotoxicity of epithelial cells by *H. pylori*.

In the same way, Mandalari et al. [17] recapitulated existing knowledge on citrus fruits and their bioactive compounds against *H. pylori*, and thus, the study indicated that β-Myrcene, hesperetin-7-O-glucoside, boropinic acid, sudachitin, 3′-demethoxysudachitin, auraptene, and bergamottin reduced the viability of *H. pylori* to 90%. Thereby, the polyphenols of citrus fruits and their derivatives produce an effective inhibition and eradication of *H. pylori*, and these positive effects can be individually or in combination with various antibiotics. In the same way, Takeuchi et al. [89] reported the constituents derived from nature to counteract the negative effects of *H. pylori* specie. Thus, the authors mentioned the potential of bioactive compounds found in foods and their derived products such as bovine milk (lactoferrin), green tea (catechin), broccoli sprout (sulforaphane), garlic (allicin), ginger (6-shogaol, 6-gingerol, 8-gingerol, and 10-gingerol), and apple peel (quercetin glycosides), among others, as alterative agents instead of antibiotics, and in turn, these foods can be more effective and safe to the consumers, helping in the use of a low dose of antibiotics. Specifically, Escandón et al. [90] reported that kaempferol and (-)-epicatechin had a significant suppression effect on the growth of *H. pylori* in a concentration-dependent manner. The results also proved that the inhibitory effect of *H. pylori* depends on the time of exposure to polyphenol accumulation. Thus, a mixture of low doses of kaempferol and (-)-epicatechin exhibited antibacterial activity. Chua et al. [91] explored the effects of blueberry and grape seed extracts for *H. pylori* eradication, showing no significant difference between the extracts and placebo group to eradicate the pathogen. However, the study emphasizes that the procyanidins from grape seed can help as an anti-inflammatory adipokine on low-grade inflammatory diseases. Cardoso et al. [92] studied Wild strawberry leaves (*Fragaria vesca* L.) and Agrimonia eupatoria L. (plant of the *Rosaceae* family) extracts, and an ellagitannin-enriched fraction of wild strawberry leaves against different clinical isolates of *H. pylori*, where all the fractions presented anti-*H. pylori* activity, but the results revealed an intense activity of ellagitannin-enriched fraction of wild strawberry leaves to prevent the growth of *H. pylori* infection, since it inhibited 67% of the pathogen, suggesting potential of plant extracts as bioactive agents in association with antibiotic therapy. Park et al. [93] used red raspberry (*Rubus crataegifolius*) and elm tree (*Ulmus macrocarpa*) to counteract the presence of *H. pylori* in the human body, and the study showed that the ellagic acid of red raspberry and the catequin of elm tree achieved excellent results against the pathogen, since these bioactive compounds prevented (separately or in combination) the growth of *H. pylori*. Thereby, the combination of red raspberry (75 μg/mL) and elm tree (75 μg/mL) proved to have a synergetic effect against this pathogen. Thereby, the combination of samples could be an alternative as a new and safe herbal product against anti-*H. pylori* infection. Moreover, Torres et al. [94] demonstrated that proanthocyanidins of avocado significantly decreased inflammatory signals of gastric adenocarcinoma cells infected with *H. pylori*, since it decreased the adherence of *H. pylori* infection in the organism. Similarly, Spósito et al. [95] indicated that *Caseria sylvestris* leaves derivatives displayed an important anti-*H. pylori* activity, where terpenes (diterpenes and sesquiterpenes), phenolic compounds presented high synergism against *H. pylori* through in vitro and in vivo assays. Betoret et al. [96] developed a low moisture apple snack impregnated with mandarin and pineapple/grape juices, and inoculated with a high microbial content of *Lactobacillus salivarius* spp. *salivarius*. Thus, the snacks showed positive effects against *H. pylori*, since the polyphenols and pH of the juices impeded the growth of the pathogen, and in turn, this combination allowed the growth of the probiotic. Barrera et al. [97] evaluated the effects of phenolic compounds (vitamin C, total phenols and flavonoids) and antioxidant content (ABTS-TEAC and DPPH assays) of clementine juice inoculated with *Lactobacillus salivarius* spp. *salivarius* on microbial counts and survival to in vitro digestion, where the results indicated that the inhibition of *H. pylori* growth was produced in all juices. Recently, Gao et al. [98] studied the effect of cranberry juice consumption (240 and 480 mL of juice, and 480 mL of placebo) on oxidative stress biomarkers and patients affected by *H. pylori* infection. The blood results indicated that cranberry juice remodeled the gut microbiota of the hospitalized patients, since the juice decreased the growth of *H. pylori*. Furthermore, Jahan et al. [99] revealed the opportunity to use pongamol extract, a flavonoid derivative present in *Pongamia pinnata* and *Tephrosia purea* against *H. pylori*, since this herbal exhibits diverse pharmacological activities, and thus, pongamol extract can be considered as an herbal medicine. Mishra et al. [100] demonstrated the inhibition of *H. pylori* infection through rosmaric acid of Asian Basil (*Ocimum* spp.), and other studies mentioned a relationship between *H. pylori* and the consumption of vegetables, since a lifestyle without dietary vegetables such as sprouted peas, Chinese chive (*Allium tuberosum*), and olive oil increase the susceptibility to contract *H. pylori* infection [39,101,102]. Table 2 shows the most relevant studies on juices, plant extracts, and certain polyphenols with anti-*H. pylori* properties.

## 5. Impact of Polyphenols on the Composition of Gut Microbiota

The gut microbiota is a complex microbial ecosystem that interacts with the human host. Gut microbiota plays an important role in nutrient regulation and various metabolic pathways and in maintaining the immune system and general health condition of the host [103]. The age, diet as well as the use of antibiotics and consumption of pre-and probiotics can affect the gut microbiota composition. Increasing evidence strongly suggests that besides dietary fibers, other foods like polyphenols are also considered as prebiotics [104].

In the last decade, it has been established that some polyphenols influence the gut microbiota [105], inhibiting the growth of some bacteria, while other bacteria can become activated [106]. Therefore, Morais et al. [107] reported that the consumption of polyphenols improved the growth and establishment of the *Bifidobacteriaceae* and *Lactobacillaceae* families and inhibited pathogenic bacteria such as *E. coli*, *Clostridium perfringens*, and *H. pylori*. The interaction between polyphenols and the gut microbiota can be approached both from the perspective of how they are metabolized by the microbiota and how they can modulate the microbiota [108,109]. Therefore, evidence indicates that the consumption of polyphenols contributes to maintaining intestinal health through their stimulating effects on the growth of beneficial bacteria and the inhibition of pathogenic bacteria, similar to prebiotics [110].

On the other hand, the consumption of polyphenols in a normal diet is accompanied by other food components that influence the composition of gut microbiota, such as fat and fiber [72]. In this context, Havlik et al. [111] reported a possible beneficial effect on health in the interaction of dietary fibers (pectin, psyllium and resistant maltodextrin) with rutin (a flavonoid), changing the levels of short-chain fatty acids and phenolic acids.

As mentioned above, the composition of the intestinal microbiota influences the health of the host, so that unfavorable changes can allow the colonization of abnormal microorganisms in the intestine that can lead to dysbiosis. This can cause different-nature immune and inflammatory responses, low-grade inflammation, weight gain, adiposity, and other metabolic problems [21,107]. Thus, Kemperman et al. [106] reported that black tea polyphenols produced a decrease in the concentration of beneficial *Bifidobacteria* and an increase in pathogenic bacteria such as *Klebsiella*, a change that slowed the host’s overall metabolism. However, most researchers show that the consumption of polyphenols causes favorable changes in the gut microbiota, increasing the acidolactic bacteria mentioned above. This modulation of the composition of the gut microbiota produces an increase in the composition of short-chain fatty acids, a decrease in obesity, inflammation, and the metabolism of adipogenesis and lipogenesis [72].

In general, it is complex to determine the direct effects of polyphenols on the intestinal microbiota, since they are highly variable depending on the source, chemical nature, and dose of the polyphenol [110]. However, Ma and Chen [51] recommended a daily intake of 396 mg of polyphenols for optimal human health. From a scientific point of view, it is not yet possible to report the precise mechanisms by which polyphenols modulate the intestinal microbiota, mainly due to the enormous chemical diversity of polyphenolic compounds [108,109].

## 6. Concluding Remarks and Future Perspectives

*H. pilory* infection is one of the main causes of gastric cancer and other diseases, and it has become increasingly vital research to treat due to antibiotic resistance. Consequently, polyphenols may contribute to the gastrointestinal health, since these bioactive compounds can prevent the proliferation of *H. pylori*. Also, polyphenols can modulate the gut microbiota and decrease oxidative stress, and thus, polyphenols improve the body’s immune system. The microbiota impacts the biotransformation of polyphenols into low-molecular-weight, allowing a high absorption, transport and delivery to the epithelium, ending in a correct distribution in the bloodstream, tissues, and brain, of the host. Thus, a stable synergy between the microbiota and polyphenols is essential to maintain the balance. Non-traditional therapies are required to address the pathogens antibiotic resistance, especially for *H. pilory* infection. Therefore, it is essential to continue the evaluation of dietary polyphenols from natural sources as a new and novel approach, especially endemic berries fruits with high amount of polyphenols content and antioxidant activity such as blueberry, calafate, murta, and maqui, among others, allowing to improve the microbiota and immune response against infections. Many studies have focused on a polyphenol or a mixture of a number of bioactive compounds, with anti-*H. pylori* properties. Thereby, the bioactive components of fruits, vegetables, and plants (stems, leaves, seeds, etc.), should be isolated, identified, and tested separately to understand the action mechanism in the human body as well in pathogens, and in turn, these components could be used as a new and safe product, and novel alternative therapies to eradicate the *H. pylori* infection.

## Figures and Tables

**Table 1:** Biotransformation of polyphenols by gut microbiota and main metabolites (Adapted from Marin et al. [76]).

Polyphenol Group	Compound	Gut Microbiome	Metabolites
Anthocyanins	Cyanidin	*Bifidobacterium lactis* BB-12	3,4-Dihydroxybenzoic Acid
	Malvidin	*Lactobacillus acidophilus* LA-5	3,4-Dimethocybenzoic Acid
	Ponidin Pelargonidin	*Lactobacillus casei* *Lactobacillus plantarum*	3-Methoxy-4-Hydroxybenzoic Acid 4-Hydroxybenzoic Acid
Flavan-3-ols	Catechin	*Clostridium cocoides*	3-(3-Hydroxyphenyl)-Propionic Acid
	Epicatechin Epigallocatechin	*Bifidobacterium* spp.	5-(3′,4′-Dihydroxyphenil)-Γ-Valerolactone 5-(3,4-Dihydroxyphenyl)-Valeric Acid 3-(3,4-Dihydroxyphenyl)-Propionic Acid
			5-(3′,4′-Dihydroxyphenyl)-Γ-Valerolactone 5-(3′,5′-Dihydroxyphenyl)-Γ-Valerolactone
Flavanones	Naringenin	*Clostridum* Strains	3-(4-hydroxyphenyl)-propionic acid
		*Eubacterium ramulus*	
Flavones	Luteolin	*Clostridium orbiscindens*	3-(3,4-Dihydroxyphenyl)-Propionic Acid
	Apigenin	*Enterococcus avium*	3-(4-Dihydroxyphenyl)-Propionic Acid 3-(3-Dihydroxyphenyl)-Propionic Acid 4-Hydroxycinnamic Acid
Flavonols	Kaempferol	*Clostridium orbiscindens*	2-(4-Hydroxyphenyl)-Propionic Acid
	Quercetin Myricetin	*Clostridium orbiscindens* *Eubacterium oxidoreducens* *Eubacterium ramulus* *Enterococcus casseliflavus* *Clostridium orbiscindens* *Eubacterium oxidoreducens*	2-(3,4-Dihydroxyphenyl)-Acetic Acid 2-(3-Hydroxyphenyl)-Acetic Acid 3-(3,4-Dihydroxyphenyl)-Propionic Acid 3-(3-Hydroxyphenyl)-Acetic Acid 2-(3,5-Dihydrosyphenyl)-Acetic Acid 2-(3-Hydroxyphenyl)-Acetic Acid
Isoflavones	Daidzein	*Bacteroides ovatus*	(S)-Equol
		*Streptococcus intermedius* *Rumnococcus products* *Eggerthella* sp. Julong 732 *Enterococcus faecium* EPI1 *Lactobacillus mucosae* EPI2 *Finegoldia magna* EPI3 *Clostridium* sp. HGHA136	O-Demethylangolesin

**Table 2:** Juices, plant extracts, and polyphenols with anti-*H. pylori* effects.

Sample	Test Type	Anti-*Helicobacter pylori* Effect	Reference
Bergamot Juice	In vitro	Inhibited the Growth and Reduced the Viability	[17]
Citrus Fruit Extract	In vitro	Antimicrobial Activity, Affecting the Urease Activity	[17]
6-Shogaol, 6-Gingerol, 8-Gingerol 10-Gingerol Curcumin Propolis Muscadine Grapes	In vitro	Antibacterial Activity Inhibited the Growth and Damaged Its Cytoplasmic Membrane	[89]
Kaempferol And (-)-Epicatechin	Inhibition Halo Test	Suppressed the Growth, also proved the Inhibitory Effect depends on the Pathogen Exposure Time to the Polyphenol	[90]
Blueberry and Grape Seed Extract	Standard Triple Therapy Plus	There was no Significant Difference between this Therapy and one Standard Triple Therapy Plus Placebo	[91]
Aqueous Extract from *Fragaria Vesca* Leaves	Antibacterial Activity Evaluation	Revealed an Intense Activity Due to the Source of Ellagitannins	[92]
Red Raspberry Elm Tree	In vitro	Inhibited (Alone or in Combination) the Growth of *H. Pylori*	[93]
Avocado	Anti-Inflamatory Activity	Lowered the Early Inflammatory Signals	[94]
*Caseria Sylvestris* Leaves	In vitro and in vivo	Inhibited the Growth of *H. Pylori*	[95]
Low Humidity Apple Snack Impregnated with Mandarin and Pineapple/Grape Juice, and Inoculated with *Lactobacillus Salivarius* spp. *Salivarius*	In vivo	No Evidence Suggested Whether Bioactive Compounds Present in the Juices Affected the Eradication Rate	[96]
Clementine Juice Inoculated with *Lactobacillus Salivarius* spp. *Salivarius*	In vitro	Inhibited the Growth of *H. Pylori*	[97]
Cranberry Juice	Standard Triple Therapy Plus	Inhibited the Growth of *H. Pylori*	[98]

## Data Availability

Not applicable.
